# Unusual Presentation of Epidermodysplasia Verruciformis (EV) in Non-Sun Exposed Area: A Case Report

**DOI:** 10.1155/crdm/7801944

**Published:** 2025-03-26

**Authors:** Nidal Jebrini, Majed Dwaik, Mohanad Jaber, Sami Jabari, Raghad Razem, Man Sarahna, Rashad Alzaro, Feras Aljabari, Mohamed Aqel, Husein Sarahneh

**Affiliations:** ^1^Hemato-Oncology Department, Faculty of Medicine, Palestine Polytechnic University, Hebron, State of Palestine; ^2^Dean's Office, Faculty of Medicine, Palestine Polytechnic University, Hebron, State of Palestine; ^3^Forensic Medicine Department, Faculty of Medicine, Palestine Polytechnic University, Hebron, State of Palestine; ^4^Faculty of Medicine, Palestine Polytechnic University, Hebron, State of Palestine; ^5^Dermatology Department, Al-Ahli Hospital, Hebron, State of Palestine; ^6^Plastic and Reconstructive Surgery Department, Al-Ahli Hospital, Hebron, State of Palestine; ^7^Pathology Department, Al-Ahli Hospital, Hebron, State of Palestine; ^8^Internship Program, Princess Alia Governmental Hospital, Hebron, State of Palestine

**Keywords:** epidermodysplasia verruciformis, non–sun-exposed area, squamous cell carcinoma

## Abstract

**Introduction and Importance:** Epidermodysplasia verruciformis (EV), a rare hereditary skin disorder linked to HPV immunity, increases the risk of squamous cell carcinoma (SCC), typically in sun-exposed areas. This case highlights an extraordinary instance of SCC in a Sun-shielded region, marking the second documented case globally.

**Methods:** The medical records and histopathological slides of the case were retrospectively reviewed. This work has been reported based on the CARE criteria.

**Case Presentation:** A 28-year-old Palestinian woman, who adheres to a sun-protective Hijab due to her Muslim faith and has limited sun exposure working in a clothing store, with painful scalp lesions presented at the dermatology clinic. She and her siblings were diagnosed with EV. Three years ago, a painful, enlarging lesion on her scalp led to a diagnosis of trichoblastic carcinoma, followed by the development of six similar lesions. A year later, she returned with multiple painful, pus-producing lesions exhibiting features of trichoblastic and verrucous carcinoma, posing a challenging clinical scenario.

**Clinical Discussion:** EV is a rare genetic skin disorder linked to EVER1/TCM6 or EVER2/TCM8 gene mutations, causing widespread warts due to specific HPV types. It heightens the risk of nonmelanoma skin cancer (NMSC), mainly SCC, often associated with beta-HPVs 5 and 8. Notably, atypical cases challenge the sun-exposure SCC concept. The reatment involves UV protection, retinoids, and close monitoring, critical to prevent lesion recurrence and aggressive malignancy interventions upon therapy discontinuation.

**Conclusion:** In this unique case, a patient with EV developed SCC in an uncommonly sun-protected skin area, highlighting the extreme rarity of such an event within the context of this condition's complications.

**Summary**

• The occurrence of epidermodysplasia verruciformis (EV) in sun-shielded areas is exceptionally rare, with few exceptions noted in this case study.
• EV significantly heightens susceptibility to nonmelanoma skin cancer (NMSC), primarily squamous cell carcinoma (SCC).
• Early lesion identification and prompt retinoid therapy are crucial in impeding disease progression and reducing recurrence risk.

## 1. Introduction

EV is an exceptionally rare and lifelong hereditary dermatological condition that commonly presents during infancy or early childhood. This disorder disrupts the body's immune defense mechanisms, rendering it incapable of effectively guarding against specific varieties of human papillomavirus (HPV), particularly those belonging to the beta-HPV subgroup. Consequently, individuals afflicted with EV exhibit a distinctive combination of plane warts and lesions resembling pityriasis versicolor from an early age [1].

Patients with EV face a heightened susceptibility to the development of SCC, particularly in their third and fourth decades of life. Notably, these malignancies tend to manifest predominantly in sun-exposed regions of the skin, underscoring the pivotal role of ultraviolet radiation as a significant cocarcinogenic factor, in conjunction with HPV infection [2]. Within the context of EV-associated carcinomas, HPV DNA sequences, most notably HPV Types 5 and 8, consistently feature as identified pathogens within these neoplastic lesions [3]. Here, we present a rare case of a patient with EV who has developed SCC in a non–sun-exposed area of the skin.

## 2. Case Presentation

A 28-year-old Palestinian woman presented to the dermatology clinic with multiple painful, ulcerative, and purulent lesions scattered across her scalp. The lesions had developed gradually over the past year. Due to socioeconomic challenges, she had delayed seeking medical attention. As part of her religious practice, she habitually covers her scalp with a Hijab, limiting sun exposure. Additionally, her work in a clothing store confines her to an indoor environment, contributing to minimal sun exposure. However, while limited sunlight may be relevant to her condition, it is not assumed to be a primary factor in lesion development.

The patient has a notable history of EV, diagnosed at age 13 when she and her siblings presented with wart-like lesions on the head, face, and upper extremities. To manage EV-associated inflammation, she was started on isotretinoin therapy, although details of this treatment's initiation and duration were not closely documented. Prior to her current presentation, she had discontinued isotretinoin. While this discontinuation was temporally followed by an increase in scalp lesions, isotretinoin's therapeutic effect is limited and does not offer definitive prevention against malignant transformation in EV patients.

**Figure 1:**
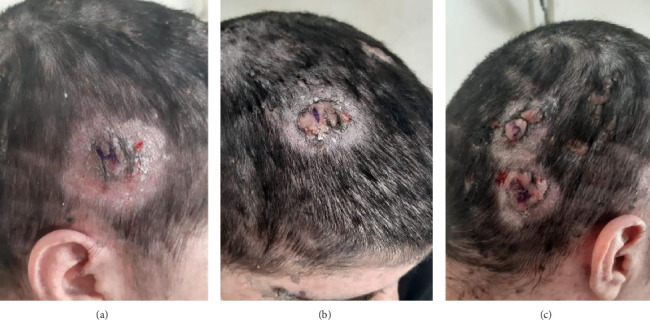
First lesions that appeared. (a) Right temporal lesion. (b) Right anterior parital lesion. (c) Superior and inferior occipital lesions.

Approximately 3 years before this presentation, one lesion on her scalp began enlarging and became painful, prompting medical evaluation. Following an initial dermatology assessment, she was referred to oncology due to suspicion of malignancy. Surgical excision of the lesion was performed by a plastic surgeon, with histopathology confirming trichoblastic carcinoma (Figure 1). Over the next several months, six additional lesions with similar characteristics appeared on her scalp, requiring further surgical intervention.

**Figure 2:**
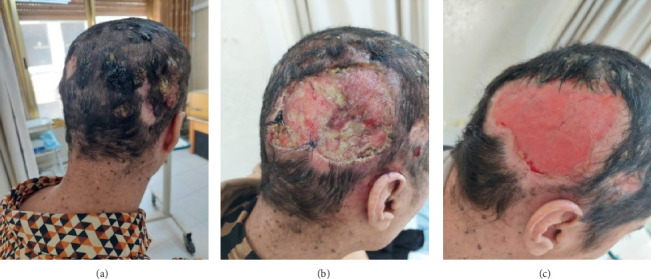
The second presentation of the lesions. (a) Three big lesions in the right occiput. (b) One week postoperation. (c) One month postoperation.

**Figure 3:**
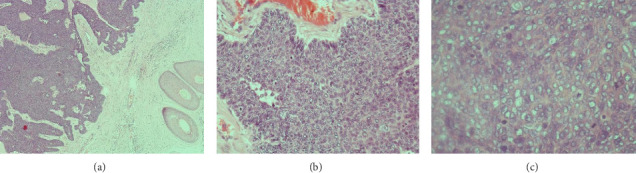
The tumor is solid and showed a multinodular growth pattern in a collagenous stromal (a). The tumor is composed of baseloid cells arranged in a palisading fashion (b). The tumor cells are large atypical with prominent nucleoli and brisk mitotic figures (c).

One year later, the patient returned to the clinic with increasing numbers of painful, pus-producing scalp lesions, primarily located in the right occipital area. Complete excision of a 6 × 9-cm region of the right occipital scalp was undertaken (Figure 2), with the specimens sent for the histopathological analysis (Figure 3). The findings revealed both trichoblastic carcinoma and verrucous carcinoma. Although scalp localization is consistent with known patterns for trichoblastic lesions, the connection between EV and lesion localization on the scalp remains speculative, given the multifactorial nature of these conditions and the need for further research.

## 3. Discussion

EV is an exceptionally rare genetic skin disorder with an autosomal recessive inheritance pattern, affecting less than one in every 1,000,000 individuals. Typically, it manifests during childhood, presenting as widespread flat warts resembling pityriasis, with an average onset age of 4.9 years [4].

The diagnosis of EV relies on clinical, histopathological, and molecular findings. Clinical features, such as the failure to eliminate lesions after therapy, and histopathological characteristics, such as gray–blue cytoplasm and enlarged nuclei, contribute to the diagnosis. Autosomal recessive abnormalities in EVER1/TCM6 or EVER2/TCM8 may cause classical hereditary EV, with these genes located on Chromosome 17 [5–7].

EV is strongly correlated with HPV, particularly beta-HPV Types 5, 8, 9, 12, 14, 15, and 17 and Types 19–25, 36–38, 47, and 50. High-risk strains such as HPV-5 and HPV-8 significantly increase the risk of NMSC [6, 8]. Mutations in TMC genes increase susceptibility to specific HPV infections, predisposing individuals to EV [8, 9]. Cutaneous malignancies occur in 30%–70% of EV cases, typically after age 30, with SCC manifesting in sun-exposed areas [10]. Our case deviates from the norm, presenting SCC in non–sun-exposed skin, a rarity with only one similar case reported [5, 6].

Our case mirrors a unique instance in a 25-year-old Iranian male, challenging the notion that SCC in EV primarily arises in sun-exposed areas. Both cases emphasized the importance of monitoring individuals with EV, irrespective of sun exposure, showcasing clinical diversity [5].

The treatment for EV centers on preventing benign lesions from progressing to malignancy. UV protection from early childhood and oral retinoids are recommended [11]. It is essential to note that the effects of retinoid therapy are often reversible upon discontinuation. These medications offer several benefits, including antiviral properties and the regulation of epithelial cell differentiation, thereby assisting in the management of disease progression [4, 12]. In our case, the patient discontinued isotretinoin, leading to lesion recurrence and necessitating multiple surgical interventions, including a 6 × 9-cm scalp excision to proactively prevent further recurrence.

## 4. Patient's Persecriptive

The patient's perspective provides a firsthand account of the challenges they have faced due to lesions on their scalp, which have gradually emerged over the past year, leading to considerable physical and emotional distress. Despite adhering to religious practices necessitating scalp coverage and predominantly working indoors, the persistence of these lesions has significantly disrupted their daily life.

Furthermore, the patient's account of a familial history involving similar lesions, initially diagnosed as EV, suggests a predisposition to cutaneous malignancies. This raises concerns regarding disease progression and complicating factors such as trichoblastic carcinoma and verrucous carcinoma, as revealed through the recent surgical intervention.

In the patient's own words: “The pain got worse, especially at night, and these lesions produce a lot of pus. Recently, they did a big surgery and found both trichoblastic carcinoma and verrucous carcinoma. Doctors explained that “It's a tough situation, but I'm hoping they'll find a way to help me feel better. Just want this pain and uncertainty to go away.”

Given the patient's poignant narrative and the complexity of their medical journey, it is imperative to delve into the long-term outcomes following surgical interventions for SCC on the scalp. This entails a thorough assessment of postoperative complications, including wound healing issues, infection, and the potential for SCC recurrence. Additionally, developing comprehensive strategies for pain management and addressing the patient's uncertainties regarding their prognosis are essential for providing holistic care and alleviating their distress.

## 5. Conclusion

In this particular case, we present an exceedingly rare occurrence wherein a patient diagnosed with EV has developed SCC in a region of the skin that is not typically exposed to sunlight. The rarity of this case underscores its exceptional nature within the context of EV-associated complications.

## Data Availability

The data that support the finding of this study are available from the corresponding author upon reasonable request.
